# Cytotoxicity of Poly(Phenolic)Sulfonates and Their Sodium Salts in L1210 Lymphoid Leukemia Cells

**DOI:** 10.1155/MBD.1998.147

**Published:** 1998

**Authors:** Fei Wang, Amy E. Warren, Cheryl R. Barnes, Iris H. Hall

**Affiliations:** Division of Medicinal Chemistry and Natural Products University of North Carolina Chapel Hill Chapel Hill N.C. 27599-7360 USA

## Abstract

Poly(phenolic)-sulfonates demonstrated very good cytotoxicity against the growth of tumor cell lines (L1210, Tmolt-_3_, HeLa-S^3^) and are comparable in potency with typical clinically used anticancer drugs. Four of the most active compounds, i.e. GL-2021, GL-2029, GL-2041 and GL-2063, were selected for a mode of action study in L1210 lymphoid leukemia cells at concentration of 25μM to 100μM for 60 min. The agents did not alkylate bases of ct-DNA, cause intercalation between base pairs, produce cross linking of ct-DNA strands or generate free radicals although L1210 DNA fragmentation was observed after 24 hr incubation. L1210 DNA synthesis was preferentially inhibited which was achieved by (1) suppressing DNA polymerase α activity which reduced the synthesis of new strands of DNA, (2) reducing of *de novo* purine synthesis at the regulatory enzyme PRPP amido transferase which reduced d(GMP) levels, and (3) inhibiting of nucleoside kinase activities which further reduced DNA synthesis. DNA template activity was altered by the poly(phenolic)sulfonates since they reduced DNA polymerase α and m-RNA and t-RNA polymerase activities. The kinetic studies at 50 μM over 2 hr demonstrated that the agents’ effect on PRPP-amido transferase activity is probably a major target of the compounds.


### CYTOTOXICITY OF POLY(PHENOLIC)SULFONATES AND THEIR SODIUM SALTS IN L1210 LYMPHOID LEUKEMIA CELLS

Fei Wang, Amy E. Warren, Cheryl R. Barnes and Iris H. Hall*

Division of Medicinal Chemistry and Natural Products
University of North Carolina Chapel Hill, N.C. 27599-7360, USA

### ABSTRACT

Poly(phenolic)-sulfonates demonstrated very good cytotoxicity against the growth of tumor cell lines (L1210, Tmolt-$_3$, HeLa-S$^3$) and are comparable in potency with typical clinically used anticancer drugs. Four of the most active compounds, i.e. GL-2021, GL-2029, GL-2041 and GL-2063, were selected for a mode of action study in L1210 lymphoid leukemia cells at concentration of 25μM to 100μM for 60 min. The agents did not alkylate bases of ct-DNA, cause intercalation between base pairs, produce cross linking of ct-DNA strands or generate free radicals although L1210 DNA fragmentation was observed after 24 hr incubation. L1210 DNA synthesis was preferentially inhibited which was achieved by (1) suppressing DNA polymerase α activity which reduced the synthesis of new strands of DNA, (2) reducing of *de novo* purine synthesis at the regulatory enzyme PRPP amido transferase which reduced d(GMP) levels, and (3) inhibiting of nucleoside kinase activities which further reduced DNA synthesis. DNA template activity was altered by the poly(phenolic)sulfonates since they reduced DNA polymerase α and m-RNA and t-RNA polymerase activities. The kinetic studies at 50 μM over 2 hr demonstrated that the agents' effect on PRPP-amido transferase activity is probably a major target of the compounds.

### INTRODUCTION

Large molecular weight polymers, e.g. sulfated polysaccharide dextran sulfate, pentosan polysulfate, have demonstrated anti-HIV-1 and anti-HIV-2 activity by interfering with binding of the gp120 protein to CD4 and blocking reverse transcriptase activity [1]. Hepsulfam, a new antineoplastic alkanesulfonate agent, has demonstrated a broad preclinical activity in human tumor xenografts [2,3] and oat cell lung cancer; however, this agent exhibited *in vitro* toxicity in human bone marrow cells [3]. Suramin, a known antitrypanosomal agent, was found to exert a strong inhibitory effect on RNA-directed DNA polymerase (reverse transcriptase) activity of several oncornaviruses such as Moloney murine leukemia virus, murine Rauscher leukemia viruses, Moloney murine sarcoma virus and avian myeloblastosis viruses [4-7]. Related to suramin is a class of sulfonic acid azo dyes, for example, Evans blue, which has been shown to be active against replication of the AIDS virus [6]. Recently a series of poly(phenolic)sulfonated compounds were shown to have potent anti-inflammatory activity and protection against induced endotoxic shock in mice at 8 and 16 mg/kg, I.P [8]. These agents prove to be potent elastase and prostaglandin cyclooxygenase inhibitors, blocking the release of TNFα and IL-1 release. The binding of these cytokines to high affinity receptors on their target cells were also competitively suppressed by the agents. These compounds blocked the adhesion of leukocytes and macrophages to L929 fibroblasts grown in tissue culture [5]. Because of these properties it was surmised that these poly(phenolic)sulfonated compounds may have antineoplastic activity.

### METHODS

### Source of Compounds

Test compounds were supplied by GeneLabs, Inc. [Redwood City, CA [Table 1]. Radioisotopes were obtained from New England Nuclear [Dupont, Boston, MA]. Substrates and co-factors were purchased from Sigma Chemical Co., [St Louis, MO]. Pentosan sulfate and other antineoplastic standards were obtained from Sigma Chemical Co. Tumor lines were obtained from American Type Culture Collection [Rockville, MD].

**Table 1 Structures of Poly(phenolic)sulfonate Compounds**

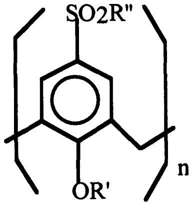

| Compound # | R' | R" | n | MW |
|---|---|---|---|---|
| Gl-2041 | H | OH | 8 | 1489.46 |
| Gl-2029 | H | OH | 6 | 1117.09 |
| Gl-2030 | H | OH | 4 | 744.73 |
| Gl-2021 | $CH_3$ | ONa | 8 | 1777.5 |
| Gl-2057 | $CH_3$ | ONa | 4 | 888.75 |
| Gl-2158 | $CH_3$ | ONa | 6 | 1333.13 |
| Gl-2063 | $OCH_3$ | ONa | 8 | 2001.65 |
| Gl-2042 | OAc | ONa | 8 | 2017.73 |

**Cytotoxicity**

Poly(phenolic)sulfonates were tested for cytotoxic activity by homogenizing drug in 0.05% Tween 80/$H_2O$ to give a 1 mg/ml stock concentration. These solutions were sterilized by passing them through an acrodisc (45 µm). The following cell lines were maintained by literature techniques [9]: murine $L_{1210}$ lymphoid leukemia, P-388 lymphocytic leukemia, rat UMR 106 osteosarcoma, human $Tmolt_3$ acute lymphoblastic T cell leukemia, HuT-8 lymphoma, HeLa-$S^3$ suspended cervical carcinoma, HeLa solid cervical carcinoma, KB epidermoid nasopharynx, A431 epidermoid carcinoma, colorectal adenocarcinoma SW480, HCT-8 ileocecal adenocarcinoma, lung bronchogenic MB-9812, A549 lung carcinoma, and glioma HS683. Geran *et al.*'s protocol [9] was used to assess the cytotoxicity of the compounds and standards in each cell line. Cell numbers were determined by the trypan blue exclusion technique. Solid tumor cytotoxicity was determined by Leibovitz *et al.'s* method [10] utilizing crystal violet/MeOH and read at 562 nm (Molecular Devices). Values for cytotoxicity were expressed as $ED_{50}$ = µg/ml, i.e. the concentration of the compound inhibiting 50% of cell growth. A value of less than 4 µg/ml was required for significant activity of growth inhibition.

**Incorporation Studies**

Incorporation of labeled precursors into $^3$H-DNA, $^3$H -RNA and $^3$H -protein for $10^6$ L1210 cells was obtained [11]. The concentration response for inhibition of DNA, RNA and protein synthesis of the compounds at 25, 50 and 100 µM was determined for 60 min incubations. Incorporation of $^{14}$C -glycine (53.0 mCi/mmol) into purines was obtained by the method of Cadman *et al.*[12]. Incorporation of $^{14}$C-formate (53.0 mCi/mmol) into pyrimidines was determined by the method of Christopherson *et al.*[13].

**Enzyme Assays**

Inhibition of various enzyme activities was performed by first preparing the appropriate L1210 cell homogenates or subcellular fractions, then adding the drug to be tested during the enzyme assay. For the concentration response studies, inhibition of enzyme activity was determined at 25, 50 and 100 µM of compounds Gl-2021, Gl-2029, Gl-2041 and Gl-2063 after 60 min incubations. DNA polymerase $\alpha$ activity was determined in cytoplasmic extracts isolated by Eichler *et al.'s* method [14]. The DNA polymerase $\alpha$ assay was described by Sedwick *et al.*[15] using $^3$H-TTP. Messenger-, ribosomal- and transfer-RNA polymerase enzymes were isolated with different concentrations of ammonium sulfate; individual RNA polymerase activities were determined using $^3$H -UTP [16,17]. Ribonucleoside reductase activity was measured using $^{14}$C -CDP with dithioerythritol [18]. The deoxyribonucleotides $^{14}$C -dCDP were separated from the ribonucleotides by TLC on PEI plates. Thymidine, TMP and TDP kinase activities were determined using $^3$H -thymidine (58.3 mCi/mmol) in the medium of Maley and Ochoa[19]. Carbamyl phosphate synthetase activity was determined with the method of Kalman *et al.* [20] and citrulline was determined colorimetrically [21]. Aspartate transcarbamylase activity was measured using the incubation medium of Kalman *et al.*[20], carbamyl aspartate was determined colorimetrically by the method of Koritz *et al.*[22]. Thymidylate synthetase activity was analyzed by Kampf *et al.'s* method [23].

**Table 2** **The Cytotoxicity of Poly(phenolic)sulfonates Using Mouse, Rat and Human Cultured Cell Lines**

**$ED_{50}$ values as μg/ml**

Mouse Rat Human--------------------------------------------------Human

| Compound | $L_{1210}$ Leuke mia | UMR 106 Osteosar coma | $Tmolt_3$ T Leuke mia | Hela-$S^3$ Uterine | Solid Hela | KB Naso pharynx | SW480 Colon | HCT-8-ileum | Lung MB9812 | Lung A549 | Glioma HS683 |
|---|---|---|---|---|---|---|---|---|---|---|---|
| GL2041 | 1.88 | 8.53 | 1.94 | 1.31 | 8.74 | 2.60 | 11.56 | 7.02 | 5.88 | 9.69 | 9.38 |
| Gl2029 | 1.52 | 8.38 | 2.24 | 1.04 | 6.97 | 5.98 | 5.96 | 8.06 | 9.68 | 8.04 | 7.58 |
| GL2030 | 1.75 | 8.29 | 2.96 | 1.31 | 6.07 | 5.68 | 1.81 | 6.56 | 6.78 | 7.44 | 6.46 |
| GL2021 | 1.38 | 5.21 | 1.73 | 2.18 | 1.97 | 11.2 | 1.32 | 7.40 | 4.18 | 8.51 | 4.58 |
| GL2157 | 1.33 | 7.29 | 2.34 | 1.69 | 6.29 | 7.28 | 4.68 | 7.38 | 9.75 | 7.62 | 7.74 |
| GL2158 | 2.25 | 7.87 | 1.32 | 2.02 | 8.16 | 3.24 | 5.52 | 8.80 | 8.53 | 7.62 | 8.37 |
| GL2063 | 2.75 | 8.61 | 0.71 | 1.58 | 6.18 | 3.42 | 6.77 | 8.51 | 3.49 | 10.30 | 9.47 |
| Gl2042 | 2.71 | 8.19 | 1.53 | 1.69 | 6.36 | 6.03 | 10.06 | 9.18 | 9.93 | 9.81 | 8.34 |
| Pentosan | 2.30 | 7.26 | 2.34 | 2.89 | 5.99 | 4.85 | 9.17 | 9.46 | 2.59 | 7.32 | 7.48 |
| | | | | | | | | | | | |
| 6-MP | 2.43 | 9.13 | 1.62 | 2.12 | 5.61 | 11.04 | 3.61 | 1.15 | 4.29 | 4.71 | 4.46 |
| Ara-C | 2.43 | 0.86 | 2.67 | 2.13 | 4.74 | 2.84 | 3.42 | 2.54 | 6.16 | 6.28 | 1.88 |
| Hydroxyurea | 2.67 | 2.87 | 4.47 | 1.96 | 8.12 | 5.27 | 7.33 | 1.77 | 7.18 | 8.89 | 2.27 |
| 5-FU | 1.41 | 3.52 | 2.14 | 2.47 | 4.11 | 1.25 | 3.09 | 1.12 | 5.64 | 3.58 | 1.28 |
| VP-16 | 1.83 | 3.57 | – | 7.87 | 3.05 | 3.32 | 3.34 | 3.78 | 3.50 | 4.74 | 2.44 |
| | | | | | | | | | | | |

Data from the cytotoxic assay are presented in Table 2 as $ED_{50}$ values in μg/ml which were estimated from a plot of the log concentration vs average percent inhibition of growth at each concentration where N =4.

The $^3H_2O$ measured was proportional to the amount of TMP formed from $^3$H-dUMP. Dihydrofolate reductase activity was determined by the spectrophotometric method of Ho *et al.*[24]. PRPP amidotransferase activity was determined by Spassova *et al.'s* method [25]. IMP dehydrogenase activity was analyzed with 8-[$^{14}$C]-IMP (54 mCi/mmol) (Amersham, Arlington Heights, IL) after separating XMP on PEI plates (Fisher Scientific) by TLC [26]. Protein content was determined for the enzymatic assays by the Lowry technique.

**DNA Studies**

After deoxyribonucleoside triphosphates were extracted [27], pool levels were determined by the method of Hunting and Henderson [28] with calf thymus DNA, *E. coli* DNA polymerase I, non-limiting amounts of the three deoxyribonucleoside triphosphates not being assayed, and either 0.4 mCi of ($^3$H-methyl)-dTTP or (5-$^3$H)-dCTP. The effects of poly(phenolic)sulfonates on L1210 DNA strand scission was determined by the methods of Suzuki *et al.*[29], Pera *et al.*[30], and Woynarowski *et al.*[31]. L1210 lymphoid leukemia cells were incubated with 10 µCi thymidine methyl-$^3$H, 84.0 Ci/mmol for 24 hr at 37°C. L1210 cells ($10^7$) were harvested and then centrifuged at 600 g X 10 min in PBS. They were later washed and suspended in 1 ml of PBS. Lysis buffer (0.5 ml; 0.5 M NaOH, 0.02 M EDTA, 0.01% Triton X-100 and 2.5% sucrose) was layered onto a 5-20% alkaline-sucrose gradient (5 ml; 0.3 M NaOH, 0.7 KCl and 0.01 M EDTA); this was followed by 0.2 ml of the cell preparation. After the gradient was incubated for 2.5 hr at room temperature, it was centrifuged at 12,000 rpm at 20°C for 60 min (Beckman rotor SW60). Fractions (0.2 ml) were collected from the bottom of the gradient, neutralized with 0.2 ml of 0.3 N HCl, and measured for radioactivity. Thermal calf thymus DNA denaturation studies, U.V. absorption studies and DNA viscosity studies were conducted after incubation of poly(phenolic)sulfonates 100 µM at 37°C for 24 hr [32]. Free radical generation by the poly(phenolic)sulfonates from 25 µM to 100 µM incubated for 15 min at 37°C with $10^6$ L1210 lymphoid leukemia cells was determined with 0.25 mg/ml o-phenanthroline, 0.1 mM EDTA and 0.1 mM ferric chloride in 20 mM phosphate buffer, pH 7.4 at 510 nm [33]. Superoxide scavenger activity was determined in an analogous manner with 0.4 mM ferricytochrome C in 20 mM phosphate buffer pH 7.4 at 37 °C at 550 nm.[34]. Standards, i.e. 3% hydrogen peroxide and zymosan at 5mg/ml [Sigma] were used to generate free radicals in the assays for comparison to the poly(phenolic)sulfonates.

**Statistical Test**

In Tables 3-6, the data is represented as the average of the percent of control and the standard deviations. In Fig 2-12 , all standard deviations are within 5.2% of the value. The probable significant different [*p*] between the treated raw data and the control raw data for each assay was determined using the Student's "t" test.

**RESULTS**

The poly(phenolic)sulfonates demonstrated potent cytotoxic activity [Table2] with $ED_{50}$ values of less than 4 µg/ml for significant activity. Mouse L1210 lymphocytic leukemia, human $Tmolt_3$ T cell leukemia and human HeLa-S$^3$ uterine carcinoma growth were significantly reduced by all of the agents including the standard pentosan sulfate with $ED_{50}$ values < 3 µg/ml. Gl-2157 and Gl-2021 demonstrated the best activity in the L1210 lymphoid leukemia screen. Gl-2063 was very potent in suppressing growth of human $Tmolt_3$ T cell leukemia cells. Gl-2029 demonstrated the best activity against the growth of HeLa-S$^3$ uterine carcinoma. Human solid HeLa uterine carcinoma growth was significantly reduced by Gl-2021 with an $ED_{50}$ value of 1.97 µg/ml. KB nasopharynx growth was reduced by Gl-2041 [$ED_{50}$ = 2.60 µg/ml] and moderately reduced by Gl-2158 and Gl-2063 with values of 3.24 and 3.42 µg/ml, respectively. Human SW480 adenocarcinoma colon growth was significantly reduced by Gl-2030, and Gl-2021 with $ED_{50}$ values less than 2 µg/ml. Human lung MB9812 bronchogenic growth was suppressed moderately by Gl-2063 and pentosan sulfate. Human HCT-8 ileum adenocarcinoma, lung A549, glioma HS683 and rat UMR-106 osteosarcoma growth was not affected by the test compounds.

Compounds Gl-2021, Gl-2029, Gl-2041 and Gl-2063 demonstrated the most potent activity in the mouse L1210 lymphocytic leukemia screen and consequently were selected for the mode of action study. These four agents preferentially affected L1210 lymphoid leukemia DNA synthesis with reduction of greater than 60% at 100 µM over 60 min [Tables 3-6]. RNA and protein syntheses were more moderately affected by the agents with 13 -22% reduction of RNA synthesis over 60 min at 100 µM and 29% to 43% reduction of protein synthesis over 1 hr. DNA polymerase α activity was reduced 33% to 56% after 1 hr at 100 µM. m-RNA polymerase activity was reduced 50% to 60% and t-RNA polymerase activity was reduced 24% to 41% at 100 µM over 1 hr. r-RNA polymerase activity was elevated slightly by Gl-2021 20% to

23% after 1 hr from 25 to 100 μM, but the other three agents had no significant effect on this enzyme activity.

**Table 3 Effects of Gl-2021 on L1210 Leukemia Cell Metabolism over 60 Minutes**

| N=6 | Percent of Control (X ± SD) | | | |
|---|---|---|---|---|
| Assay | Control | 25 μM | 50 μM | 100μM |
| DNA synthesis | 100±5[a] | 105±6 | 77±5* | 39±5* |
| RNA synthesis | 100±6[b] | 109±6 | 94±6 | 78±4* |
| Protein synthesis | 100±5[c] | 80±4* | 64±4* | 59±3* |
| DNA polymerase α | 100±6[d] | 76±5* | 55±4* | 52±4* |
| mRNA polymerase | 100±7[e] | 99±5 | 74±4* | 40±4* |
| rRNA polymerase | 100±4[f] | 122±7 | 120±5 | 123±4 |
| tRNA polymerase | 100±7[g] | 98±5 | 85±5 | 76±4* |
| Ribonucleoside reductase | 100±5[h] | 158±6* | 124±5 | 91±5 |
| Dihydrofolate reductase | 100±5[i] | 97±4 | 79±4* | 76±4* |
| Purine *de novo* synthesis | 100±5[j] | 121±6 | 118±4 | 74±6* |
| PRPP amido transferase | 100±6[k] | 62±5* | 61±4* | 39±4* |
| IMP dehydrogenase | 100±5[l] | 124±4 | 97±4 | 77±4* |
| Pyrimidine *de novo* synthesis | 100±5[m] | 98±6 | 94±4 | 88±5 |
| Carbamyl phosphate synthetase | 100±7[n] | 94±5 | 89±5 | 62±4* |
| Aspartate transcarbamylase | 100±6[o] | 101±6 | 100±5 | 98±4 |
| Thymidylate synthetase | 100±5[p] | 97±6 | 88±5 | 82±4 |
| Thymidine kinase | 100±6[q] | 112±7 | 96±5 | 76±4* |
| Thymidine monophosphate kinase | 100±7[r] | 110±6 | 35±4* | 32±3* |
| Thymidine diphosphate kinase | 100±6[s] | 32±4* | 32±3* | 26±3* |
| d(ATP) | 100±5[t] | | | 65± 4 |
| d(GTP) | 100±6[u] | | | 79±4 |
| d(CTP) | 100±5[v] | | | 67±5 |
| d(TTP) | 100±4[w] | | | 88±6 |

Control values for $10^6$ cells/hr *$P \leq 0.001$ Student's "t" test
a = DNA Synthesis = 26152 dpm
b = RNA Synthesis = 4851 dpm j = Purine synthesis = 92551 dpm
c = Protein Synthesis = 7461 dpm k = PRPP-amido transferase =0.121 OD units
d = DNA polymerase α = 47804 dpm l = IMP dehyrogenase = 19758 dpm
e = m-RNA polymerase = 1502 dpm m = Pyrimidine synthesis = 76058 dpm
f = r-RNA polymerase = 4239 dpm n = Carbamyl phosphate synthetase = 0.392 mol citrulline
g = t-RNA polymerase 6400 dpm o = Asparate transcarbamylase = 1.064 mol N-carbamyl aspartate
h = Ribonucleoside reductase = 2744 dpm p = Thymidylate synthetase= 18463 dpm
i =Dihydrofolate reductase= 0.868 OD units q = Thymidine kinase = 1317 dpm r = Thymidine monophosphate kinase = 1179 dpm s = Thymidine diphosphate kinase = 1891 dpm
t = d[ATP] = 6.17 pmol; u = d[GTP] = 5.27; pmol; v = d [CTP] = 6.87 pmol;w = d[TTP] = 6.94 pmol

Ribonucleoside reductase activity was not reduced by any of the four poly(phenolic)sulfonates over 1 hr but rather it was stimulated significantly particularly at the lower concentrations after this time period. Dihydrofolate reductase activity was reduced only by compounds Gl-2021 by 24% and Gl- 2063 by 46% at 100 μM after 1 hr. L1210 *de novo* purine synthesis was suppressed 18% to 46% at 100 μM of poly(phenolic)sulfonates over 1 hr with the activities of regulatory enzyme PRPP amido transferase activity being suppressed 61% to 90% and IMP dehydrogenase activity being reduced 23% to 34% at 100 μM over 1 hr. In all three of these assays the agents demonstrated a concentration dependent suppression of activity. *De novo* pyrimidine synthesis was not significantly reduced by the agents at these concentrations for 1 hr. Carbamylate phosphate synthetase activity was reduced 27% to 38% at 100 μM after 1 hr. But aspartate transcarbamylase activity was not affected by the compounds, significantly. Thymidylate synthetase activity was reduced marginally 8% to 18% at 100 μM after 1 hr. Thymidine kinase activity was suppressed 12% to 24%, TMP kinase activity was reduced 63% to 68%, TDP kinase activity was inhibited 46% to 74% after 1 hr at 100 μM. Again, the effects of the agents on these latter two enzyme activities was concentration dependent. d[NTP] pool levels were only marginally affected by the compounds. d[ATP] levels were reduced 32% to 38% after 1 hr at 100 μM; whereas, d[GTP] pools were reduced 21% to 28% by the poly(phenolic)sulfonates. d[CTP] pool levels were lowered 4% to 33% and d[TTP] levels 2% to 17% after 1 hr incubation at 100 μM. ct-DNA studies demonstrated that the agents did not affect DNA denaturation since the Tm values for the control was 82.8 °C, Gl-2021 was 81.5 °C, Gl-2029 was 82.3 °C, Gl-2041 was 83 °C and Gl-2063 was 83°C suggesting no cross linking of the strands of DNA. ct-DNA U.V. absorption from 220 to 340 nm did not demonstrate a hyperchromic shift to a higher wavelength [Fig. 1] suggesting that the agents did not interact with the individual bases of DNA, i.e. alkylation of purines and pyrimidines.

**Table 4 Effects of Gl-2029 on L1210 Leukemia Cell Metabolism Over 60 min**

| N=6 | Percent of Control (X± SD) | | | |
|---|---|---|---|---|
| Assay | Control | 25μM | 50μM | 100μM |
| DNA synthesis | 100±5 | 83±4* | 78±3* | 37±4* |
| RNA synthesis | 100±6 | 91±6 | 91±5 | 79±4* |
| Protein synthesis | 100±5 | 82±4 | 80±3* | 71±3* |
| DNA polymerase α | 100±6 | 70±3* | 60±3* | 44±3* |
| mRNA polymerase | 100±7 | 72±4* | 55±4* | 50±3* |
| rRNA polymerase | 100±4 | 115±6 | 102±4 | 107±5 |
| tRNA polymerase | 100±7 | 103±5 | 98±5 | 69±4* |
| Ribonucleoside reductase | 100±5 | 130±5* | 130±6* | 141±5* |
| Dihydrofolate reductase | 100±5 | 92±4 | 108+5 | 127±6* |
| Purine *de novo* synthesis | 100±5 | 130±6* | 97±6 | 72±4* |
| PRPP amido transferase | 100±6 | 36±4* | 30±3* | 30±2* |
| IMP dehydrogenase | 100±5 | 270±5* | 184±6* | 74±4* |
| Pyrimidine *de novo* synthesis | 100±5 | 99±6 | 96± 5 | 93± 7 |
| Carbamyl phosphate synthetase | 100±7 | 78±5* | 74±4* | 72±3* |
| Aspartate transcarbamylase | 100±6 | 100±5 | 100±5 | 98±6 |
| Thymidylate synthetase | 100±5 | 92±5 | 89±6 | 87±4 |
| Thymidine kinase | 100±6 | 105±6 | 104±6 | 76±4* |
| Thymidine monphosphate kinase | 100±7 | 118±6 | 50±5* | 32±4* |
| Thymidine diphosphate kinase | 100±6 | 57±3* | 50±4* | 27±3* |
| d[ATP] | 100±5 | | | 68±5* |
| d[GTP] | 100±6 | | | 72± 4* |
| d[CTP] | 100±5 | | | 96± 5 |
| d[TTP] | 100±4 | | | 98±5 |

ct-DNA viscosity was unchanged after incubating with agents at 100 μM for 24 hr. The standard chloroquine at 25 and 50 μM resulted in values of 262-265 sec and acriflavine at 100 μ M caused a value of 199.8 sec. and since all values, including the control, were between 217.3 and 218.6 sec., this suggests no intercalation of the poly(phenolic)sulfonates between base pairs of DNA. L1210 DNA strand scission studies after incubating 24 hr. at 100 μM [showed that DNA fragmentation occurred with smaller molecular weight DNA in the higher fractions of the gradient [Fig 2]. DNA fragmentation was more evident with Gl-2021 and the effects were more moderated with Gl-2029. Further study demonstrated that the L1210 DNA fragmentation was not caused directly by free radical generation by the agents over 15 min as determined by the Fenton reaction or the scavenger reaction [Fig 3 and 45].

Kinetic studies of L1210 lymphocytic leukemia grown in the presence of agents at 25 μM [Fig.5] and 50 μM [data not shown] over 6 days showed that the agents were cytostatic rather than cytotoxic. The concentrations employed for these studies were much higher than the concentrations for the $ED_{50}$ values from the generated cytotoxicity study. If the $ED_{50}$ values for the cytotoxic assay were converted to μM then Gl-2021 would be 0.777 μM, Gl-2029 would be 1.362 μM, Gl-2041 would be 1.263 μM and

Gl-2063 would be 1.36 μM. Nevertheless, mode of action studies are performed at higher concentrations, which are usually multiples of the ED50 values, in order to identify the targets of the agents. L1210 DNA synthesis over 2 hours in the presence of agents at 50 μM was reduced significantly after 60 min and continued to be reduced in higher magnitude throughout the 2 hr. [Fig 6] following a time dependent effect. DNA polymerase α activity was reduced after 30 min with agents at 50 μM but with Gl-2041 and Gl-2063 between 60 and 120 min the activity recovered.

**Table 5 Effects of Gl-2041 on L1210 Leukemia Cell Metabolism Over 60 Min.**

| N=6 | Percent of Control (X± SD) | | | |
|---|---|---|---|---|
| Assay | Control | 25μM | 50μM | 100μM |
| DNA synthesis | 100±5 | 98±6 | 84±5 | 33±5 |
| RNA synthesis | 100±6 | 130±5 | 94±4 | 86±4 |
| Protein synthesis | 100±5 | 79±4* | 70±3* | 70±3* |
| DNA polymerase α | 100±6 | 85±5 | 77±4* | 63±4* |
| mRNA polymerase | 100±7 | 73±4* | 65±4* | 47±3* |
| rRNA polymerase | 100±4 | 112±5 | 102±4 | 93±4 |
| tRNA polymerase | 100±7 | 108±6 | 66±4* | 60±4* |
| Ribonucleoside reductase | 100±5 | 127±5 | 127±6* | 103±4 |
| Dihydrofolate reductase | 100±5 | 105±5 | 97±4 | 92±5 |
| Purine *de novo* synthesis | 100±5 | 85±5 | 68±4* | 54+3* |
| PRPP amido transferase | 100±6 | 38±4* | 38±3* | 10±2* |
| IMP dehydrogenase | 100±5 | 131±6* | 79±4* | 70±4* |
| Pyrimidine *de novo* stnthesis | 100±5 | 112±6 | 97±5 | 95±4 |
| Carbonyl phosphate synthetase | 100±8 | 142±6* | 82±5 | 73±4* |
| Aspartate transcarbamylase | 100±6 | 106±5 | 97±4 | 94±5 |
| Thymidylate synthetase | 100±5 | 105±5 | 103±6 | 92±5 |
| Thymidine kinase | 100±6 | 116±5 | 104±5 | 79±4* |
| Thymidine monophosphate kinase | 100±7 | 80±5* | 41±3* | 36±4* |
| Thymidine diphosphate kinase | 100±6 | 89±5 | 64±5* | 54±3* |
| d[ATP] | 100 | | | 62± 5* |
| d[GTP] | 100 | | | 74±5* |
| d[CTP] | 100 | | | 89±5 |
| d[TTP] | 100 | | | 89±6 |

With two of the compounds, i.e. Gl-2021 and Gl-2029, activity remained suppressed at 60 and 90 min. [Fig 7] while with Gl-2041 and Gl-2063 the DNA polymerase α activity returned to normal at 90 min. L1210 m-RNA polymerase activity was significantly reduced after 30 min, but with increasing times of incubation achieved the lowest value , e.g. 50% to 60% at 120 min. [Fig 8 ]. PRPP-amido transferase activity was significantly reduced greater tha 60% after 60 min and remained consistently suppressed over the next 2 hr. [Fig 9]. Thymidine kinase activity was only marginally inhibited at 90 min and required 2 hr to observe at least 40% inhibition of activity [Fig 10]. TMP kinase activity demonstrated marked reduction at 60 min but the activity recovered at 90 and 120 min [Fig 11]. TDP kinase activity was erratic also with the highest reduction at 60 min but again the activity recovered after 90 min [Fig 12].

### DISCUSSION

The poly(phenolic)sulfonates like many polymeric agents, e.g. suramin, demonstrated cytotoxic activity against mouse, rat and human tumor cell lines with $ED_{50}$ values in the range of standard clinical agents. These derivatives were very effective inhibitors in suspended mouse and human tumor cells, e.g. mouse L1210 lymphoid leukemia, human $Tmolt_3$ T cell leukemia, and HeLa-$S^3$ uterine carcinoma. The compounds were not as active against the growth of solid rat and human tumor cultured cells. Selective activity was demonstrated by some of the agents. Gl-2021 was effective against human solid HeLa uterine carcinoma, Gl-2041 was active against human KB nasopharynx carcinoma, Gl-2158 was effective against lung MB9812 bronchogenic carcinoma and Gl-2030 and Gl-2021 were active against growth of human colon adenocarcinoma growth. The agents were not active against the growth of HCT-8 ileum adenocarcinoma, lung A549, nor glioma HS683 growth.

**Table 6 The Effects of GI-2063. on L-1210 Nucleic Acid Metabolism After 60 min.**

N=6 Percent of Control (X± SD)

| Assay | Control | 25μM | 50μM | 100μM |
|---|---|---|---|---|
| DNA synthesis | 100±5 | 128±7* | 89±6* | 39±4* |
| RNA synthesis | 100±6 | 94±5 | 87±4 | 87±5 |
| Protein synthesis | 100±5 | 87±6 | 80±4* | 57±4* |
| DNA polymerase α | 100±6 | 97±5 | 94±4 | 67±4* |
| mRNA polymerase | 100±7 | 129±5* | 71±5* | 45±4* |
| rRNA polymerase | 100±4 | 87±4 | 86+5 | 83±5 |
| tRNA polymerase | 100±7 | 100±5 | 70±3* | 59±3* |
| Ribonucleoside reductase | 100±5 | 128±5* | 120±5 | 112±6 |
| Dihydrofolate reductase | 100±5 | 81±6 | 81±5 | 54±3* |
| Purine *de novo* synthesis | 100±5 | 95±5 | 85±6 | 58±4* |
| PRPP amido transferase | 100±6 | 34±5* | 29±3* | 28±2* |
| IMP dehydrogenase | 100±5 | 99±5 | 96±6 | 77±4* |
| Pyrimidine *de novo* synthesis | 100+5 | 103±6 | 102±5 | 98±5 |
| Carbamyl phosphate synthesis | 100±7 | 114±5 | 105±5 | 85±5 |
| Aspartate transcarbamylase | 100±6 | 96±4 | 88±4 | 79±3* |
| Thymidylate synthetase | 100±5 | 89±4 | 87±5 | 87+5 |
| Thymidine kinase | 100±6 | 123±5* | 104±5 | 85±5 |
| Thymidine monophosphate kinase | 100±7 | 86±5 | 43±4* | 34±3* |
| Thymidine diphosphate kinase | 100±6 | 100±6 | 37±4* | 29±3* |
| d[ATP] | 100±5 | | | 68±6* |
| d[GTP] | 100±6 | | | 121±4* |
| d[CTP] | 100±5 | | | 69±5* |
| d[TTP] | 100±4 | | | 46±3* |

**Fig 1 The Effects of Poly(phenolic)sulfonates on ct-DNA Absorption From 220 to 340 nm After 24 Hours Incubation at 100 μM**

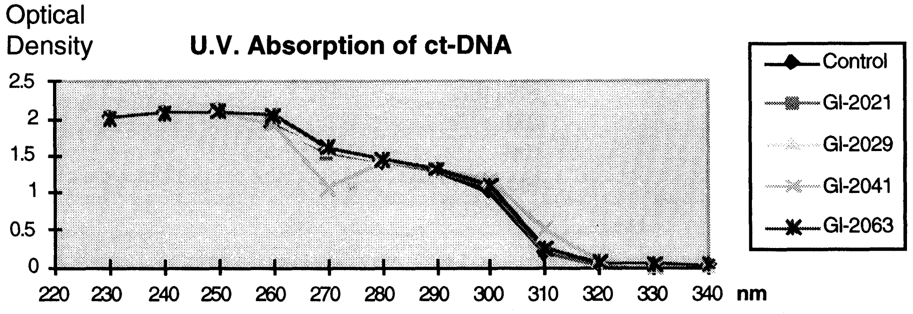

**Fig 2 The Effects of Poly(phenolic)sulfonates on L1210 Lymphoid Leukemia DNA Strand Scission at 100 μM Over 24 Hours**

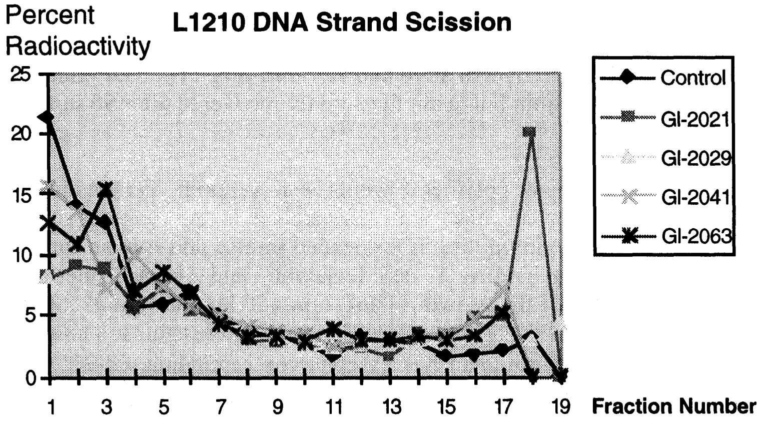

The type of selectivity demonstrated by the poly(phenolic)sulfonates, was typical of antineoplastic agents which interfere with cellular metabolism. Suramin has been shown to be an effective antineoplastic agent against Kaposi's sarcoma, non-Hodgkin lymphomas, lung carcinoma, metastatic prostate, adrenal adenocarcinoma and kidney adenocarcinoma [35-37]. Kinetic growth studies demonstrated that the poly(phenolic)sulfonate agents were cytostatic as opposed to cytocidal and they did not follow a concentration dependent inhibition of growth of L1210 cells [from 25-50 μM] at higher concentrations of the agents. This could be due to saturation of cellular transport processes by the agent or saturation of the key target receptor by the agent for inducing cell death. It is interesting to note that suramin, a hexasulfated naphthylurea, required a concentration of 250 μg/ml or 1.75 mM in HeLa cells to cause death and required 40 μM to inhibit SV40 replication in HeLa cells [38]. The growth of human lung cancers PC-9, PC-14 and H69 and their resistant strains to cisplatin or etoposide was suppressed by suramin at 160 to 400 μg/ml [39]. In Chinese hamster growth DC-3F and DC-3F /9-OH-E (resistant 9-hydroellipticine subline) $ED_{50}$ values for suramin were 31 μM and 203 μM [41].

**Fig 3 The Effects of Poly(phenolic)sulfonates on Free Radical Generation in L1210 Lymphoid Leukemia at 25-100 μM Over 15 Min**

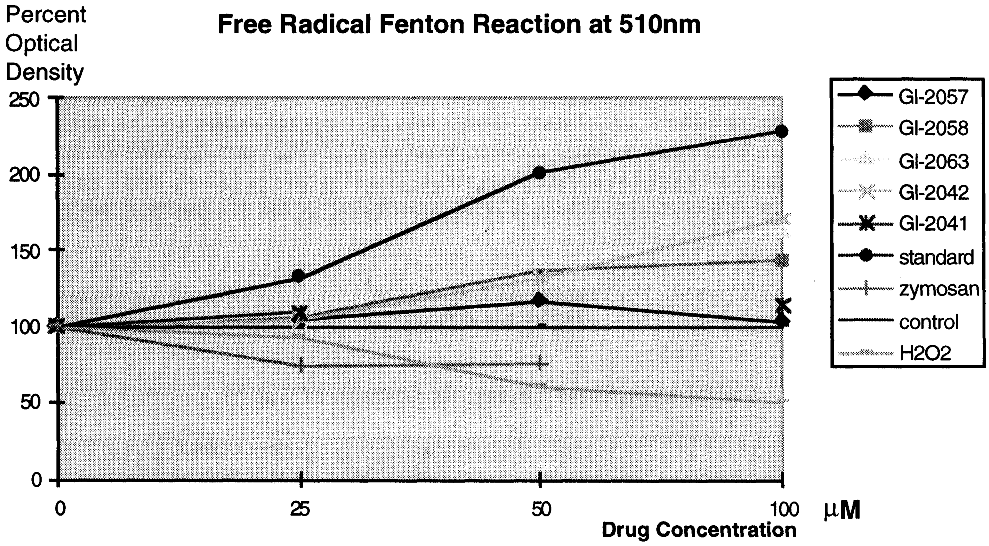

**Fig 4 The Effects of Poly(phenolic)sulfonates on Free Scavenger Generation in L1210 Lymphoid Leukemia Cells at 25-100 μM for 15 Min.**

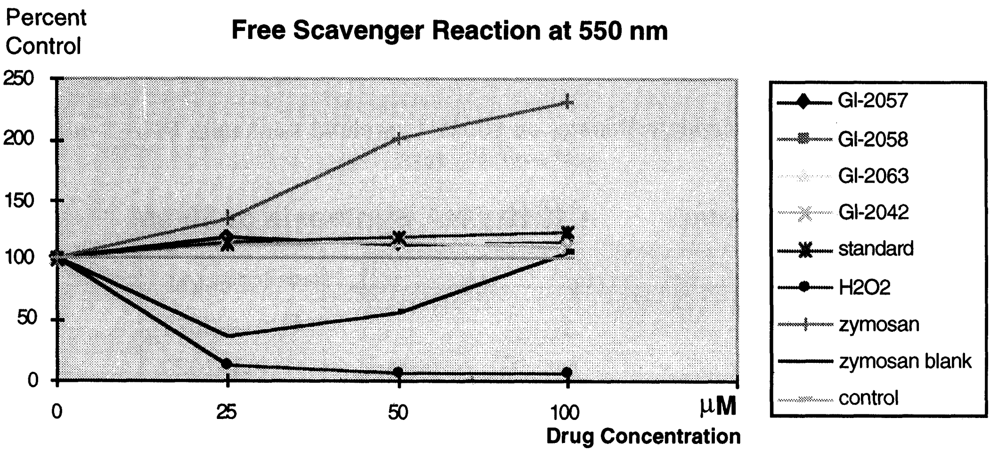

The poly(phenolic)sulfonates in the HeLa solid tumor screen afforded $ED_{50}$ values between 1.97 μg/ml and 8.74 μg/ml and the A549 and MB98 lung tumor were suppressed between 3.49 μg/ml and 10.3 μg/ml by the agents suggesting that they probably would be more potent than suramin *in vivo* in suppressing cancer growth. L1210 lymphoid leukemia mode of action studies with four of the more potent agents, i.e. Gl-2021, Gl-2029, Gl-2041, and Gl-2063 from 25-100 μM over 60 min demonstrated that DNA synthesis appears to be the major target of the agents.That is not to exclude the moderate inhibition of protein synthesis that occurred with some of the derivatives but certainly not by all four of the compounds. DNA synthesis inhibition by the poly(phenolic)sulfonates is probably the result of multiple inhibitory effects of the compounds which are additive to induce cell death. All four agents suppressed the activities of DNA polymerase α, PRPP-amido transferase and TMP and TDP kinases significantly in a concentration dependent manner. Moderate inhibition was observed by the agents for dihydrofolate reductase and IMP dehydrogenase activities. The magnitude of inhibition of either one of these enzyme activities was not sufficient to account for the observed reduction of DNA synthesis.Nevertheless, the summation of the suppression of all of these metabolic events would be sufficient to account for the observed DNA synthesis suppression in 60 min. The four agents generally followed a concentration dependent response for both the inhibition of DNA synthesis and suppression of the DNA polymerase α, PRPP-amido transferase and TMP and TDP kinases enzyme activities from 25-100 μM over 60 min. The four poly(phenolic)sulfonates demonstrated similar $IC_{50}$ values [75-84 μM] against L1210 DNA synthesis and for TMP-kinase inhibition [40-50 μM]. It did not appear that a hydroxyl group or a sodium atom in the R" affected the inhibition differently. In R' group substitutions, hydrogens, methyl or methoxy groups appeared to make little differences either. Nevertheless, all but one of the agents, i.e. Gl-2021 demonstrated a very low $IC_{50}$ value for PRPP-amido transferase inhibition [17-19 μM]. There was no obvious reason for the difference in $IC_{50}$ values of this enzyme for Gl-2021 since the only difference between Gl-2021 and Gl-2063 is that the former has a methyl group in R' and Gl-2063 has a methoxy group. The $IC_{50}$ values [21-42 μM] for TDP kinase inhibition were lower for the two compounds which were substituted in the R' position with a methyl or methoxy group.

**Fig 5 The Effects of Poly(Phenolic)sulfonates on Growth of L1210 Lymphoid Leukemia Cells at 25μM for 6 Days**

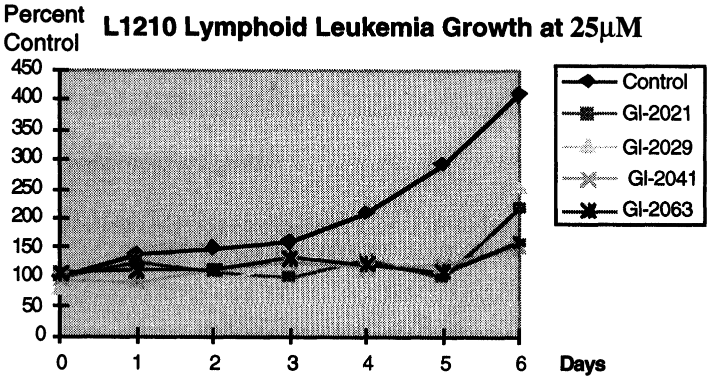

**Fig 6 The Effects of Poly(phenolic)sulfonates on L1210 Lymphoid Leukemia DNA Synthesis at 50 μM over 2 Hours**

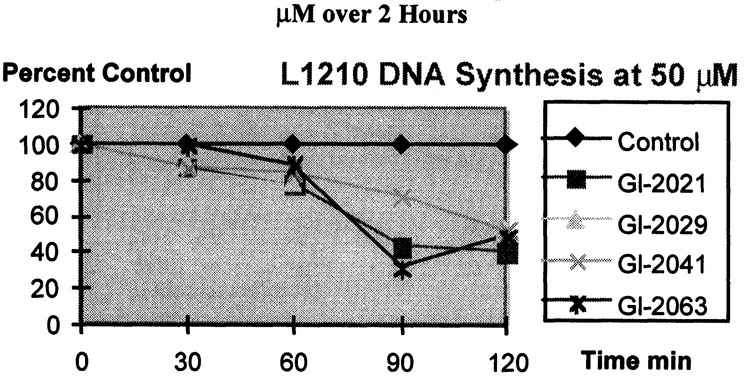

**Fig 7 The Effects of Poly(phenolic)sulfonates on L1210 Lymphoid Leukemia DNA polymerase α Activity at 50 μM Over 2 Hours**

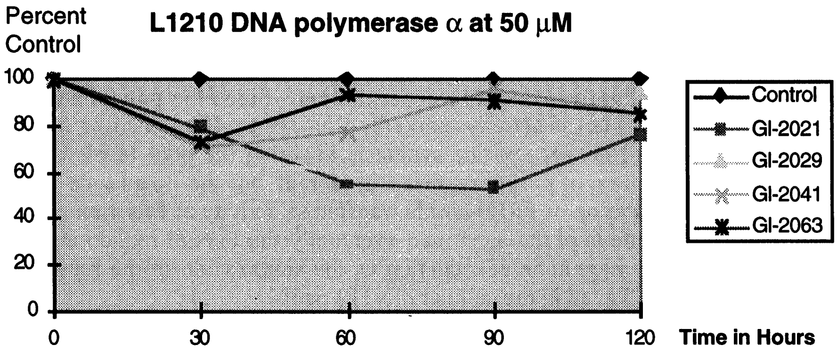

The "n" number of the compound being 6 or 8 did not seem to explain the difference observed for the $IC_{50}$ values for enzyme inhibition.

**Fig 8 The Effects of Poly(phenolic)sulfonates on L1210 Lymphoid Leukemia m-RNA polymerase Activity at 50 μM Over 2 Hours**

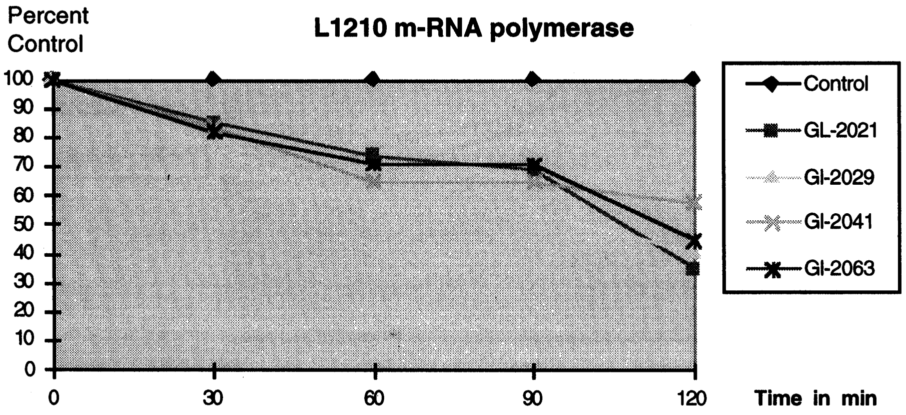

**Fig 9 The Effects of Poly(phenolic)sulfonates on L1210 Lymphoid Leukemia PRPP-Amido Transferase Activity Over 2 hours at 50μM**

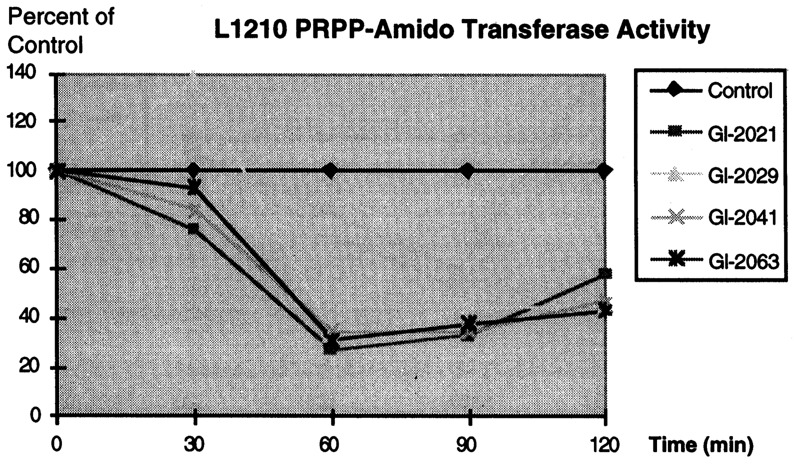

The $IC_{50}$ values [78-90 mM] for mRNA polymerase inhibition was slightly better with compounds possessing an "n" of 8. The ability of the agents to suppress multiple enzymatic sites in nucleic acid metabolism is not surprising since 6-MP, araC and 5-FU inhibit more than one metabolic process in cancer cells. Inhibition of DNA polymerase $\alpha$ activity by the agents would suppress the synthesis of a new strand of DNA in the S phase of the cell cycle. Ordinarily this would lead to the accumulation of d[NTP] pool levels. However, this was not observed after the 60 min of this study, i.e. the d[NTP] remained approximately normal or were slightly reduced in concentration. Since the poly(phenolic)sulfonates also inhibited L1210 PRPP-amido transferase activity markedly from 25 to 100 μM over 60 min, this should result in the reduction of over all *de novo* purine synthesis lowering the pool levels of AMP and GMP. These studies reflected the inhibition of purine synthesis in 60 min but the magnitude of reduction by the agent was not as great as the inhibition of PRPP-amido transferase activity at this time. This may be due simply to a time delay in the effects of the agent and eventually the overall reduction of *de novo* purine synthesis would be of the same magnitude of reduction as the suppression of the regulatory enzyme and also the effects will be further reflected in the cellular d[NTP] pools.

**Fig 10 The Effects of Poly(phenolic)sulfonates on L1210 Lymphoid Leukemia Thymidine Kinase Activity at 50 μM Over 2 Hours**

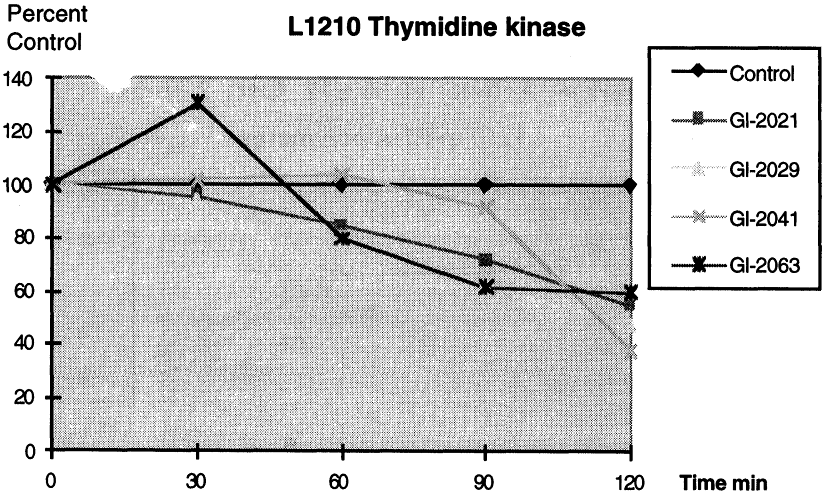

**Fig 11 The Effects of Poly(phenolic)sulfonates on L1210 Lymphoid Leukemia Thymidine Monophosphate Kinase Activity at 50 μM Over 2 Hours**

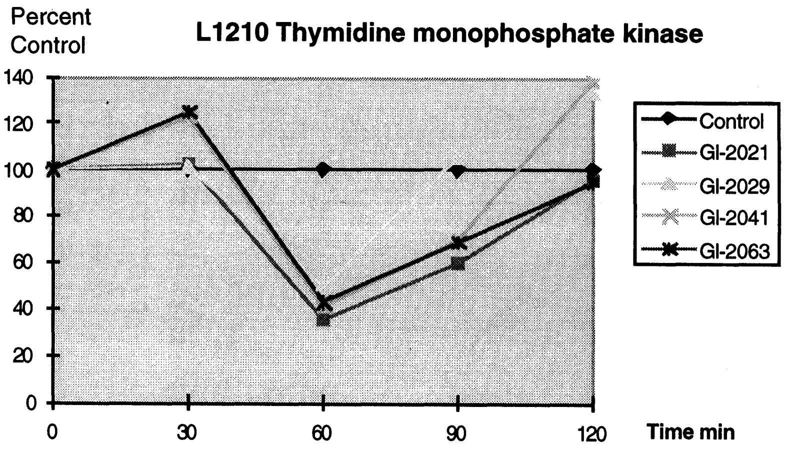

Certainly even after 60 min the purine deoxyribonucleotides were generally reduced but the effects on pyrimidine deoxyribonucleotides were not in evidence at this time of incubation. L1210 ribonucleoside reductase activity was not affected by the agents; thus, the conversion from ribonucleotides to deoxyribonucleotides was not a factor in the mode of action of these agents.

Suramin in HeLa cells inhibited DNA synthesis with an $IC_{50}$ value of 7 mM, DNA polymerase α and δ activities at 8 and 36 μM, respectively non-competitively [40] and inhibited Rauscher leukemic virus, Moloney murine sarcoma virus and avian myeloblastosis reverse transcriptase activities at concentrations at 0.1 - 1μg/ml [ 40, 7]. The viral studies suggest that suramin competes for the template binding site on the enzyme [7]. Since L1210 RNA synthesis was marginally reduced by the poly(phenolic)sulfonates over the 60 min period at 100 μM, this was probably the result of the reduction in ribonucleotides due to the reduction in *de novo* purine synthesis by these agents. This does not appear to be a major site of action of the derivatives which would be responsible for cell death or apoptosis.

**Fig 12 The Effects of Poly(phenolic)sulfonates on L1210 Lymphoid Leukemia Thymidine Diphosphate Kinase Activity at 50 μM Over 2 Hours**

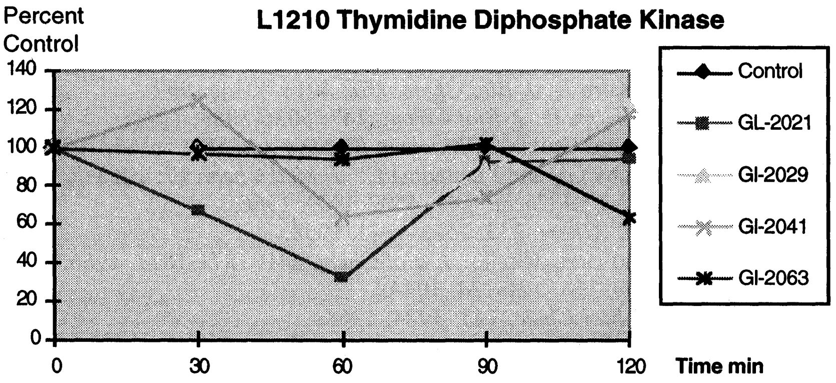

These studies also indicated that the compounds suppressed m-RNA [II] and t-RNA [III] polymerase activities after 60 min. Since in these assays all of the ribonucleotides were added to the reaction medium, this inhibition with the agents appears to be due to interference with template activity of the RNA polymerases by the compounds. Even the inhibition of DNA polymerase α activity by the agents may reflect interference with the use of the template by the poly(phenolic)sulfonates. The polyanionic nature of the agents may allow binding of some type to the DNA molecule since the histones are basic so that polymerase may be having difficulty in using the template to copy a new DNA or RNA molecules. Suramin has also been reported to block KB III nasopharynx RNA polymerase activities, *E coli* DNA polymerase I and RNA polymerase and Rauscher murine leukemia virus reverse transcriptase activity by competing for the DNA template binding site on the polymerase enzyme [42]. It was postulated that suramin binds to the basic amino acids of the enzyme perhaps via the sulfonic groups. A similar argument could be made for the observed inhibition of polymerase activities with the poly(phenolic)sulfonates. The poly(phenolic)sulfonates did not directly affect the DNA molecule nor does suramin [41]. L1210 lymphoid leukemia DNA fragmentation did occur after 24 hours incubation using whole cells. Suramin has been shown to be a DNA topoisomerase II inhibitor in PC-9 lung cancer cells and Chinese hamster fibrosarcoma cells [41]. It is possible that poly(phenolic)sulfonates function by this method also to afford DNA fragmentation. Evidence would suggest from the findings of the studies on poly(phenolic)sulfonates that they have a dual mechanism of action on cancer cells. These studies have already demonstrated that these derivatives suppress polymerase and PRPP-amido transferase activities in L1210 lymphoid leukemia cells. These studies have demonstrated that poly(phenolic)sulfonates have characteristics of the antineoplastic agents similar to suramin which has been used in advanced human malignant cancers that are refractory to standard cancer chemotherapy [43]. Whereas the studies with poly(phenolic)sulfonates are in their initial stages and certainly additional studies are warranted, and since they have demonstrated more potency in a number of these biochemical assays than suramin, they may have potential in the future as clinical agents to treat cancer patients.

**ACKNOWLEDGEMENTS**

The authors wish to thank Dr. Tom Lee of GeneLabs, Redwood City Ca, for the generous gift of the agents used in this research projects.

**REFERENCES**

1. Foye WO, Lemke Tl and Williams DA: Principles of Medicinal Chemistry. 4th edition, Williams & Wilkins, Baltimore, pp 822-845 and 871-876, 1995.
2. Eisenhauser EA and Vermorken JB: *Current Opinion Oncol.* **8**: 408-414, 1996.
3 Berger DP, Winterhalter BR, Dengler WA and Fiebig HH: *Anti-Cancer Drugs* **3**: 531-5399, 1992.
4. Eisenberg MA and Reyno LM:, *Cancer Treatment Reviews* **20**: 259-273, 1994.
5. Baumann H and Strassmann G:. *J. Immunol.* **151**: 1456-1462, 1993.
6. Hart PD and Young MR:. *Nature (Lond.)* **256**: 47-49,1975.
7. De Clercq E : *Cancer Letters* **8**: 9-22, 1979.
8. Hall IH, Murphy ME and Elkins AL: *Tissue Reaction* submitted 1997.
9. Geran RJ, Greenberg NH, MacDonald MM, Schumacher AM and Abbott BJ:. *Cancer Chemother. Rep.* **3**: 7-24, 1972.
10. Leibovitz AL, Stinson JC, McComb III WB, McCoy CE, Mazur KC and Mabry ND: *Cancer Res.* **36**: 456-459, 1976.
11. Cadman E, Heimer R, and Benz C: *J. Biol. Chem.* **256**: 1695-1704, 1981.
12. Christopherson RI, Yu ML and Jones M: *Anal Biochem.* **111**: 240-249, 1981.
13. Liao L, Kupchan SM and Horwitz SB: *Mol Pharmacol.* **12**: 167-176, 1976.
14. Eichler DC, Fisher PA and Korn D: *J. Biol Chem.* **252**: 4011-4014, 1997.
15. Sedwick WD, Wang TS and Korn D: *J. Biol Chem.* **247**: 5026-5033, 1972.
16 Anderson KM, Mendelson IS and Guizik G: *Biochem Biophys Act.* **383**: 56-66, 1975.
17. Hall IH, Carlson GL, Abernathy GS and Piantadosi C: *J. Med Chem.* **17**: 1253-1257, 1974.
18. Moore EC and Hulbert RB: *J. Biol Chem.* **241**: 4802-4809, 1966.
19. Maley F and Ochoa S: *J. Biol Chem.* **233**: 1538-1543, 1958.
20 . Kalman SM, Duffield PH and Brzozwki TJ: *J. Biol Chem.* **241**: 1871-1877, 1966.
21. Archibald RM: *J. Biol. Chem.* **156**: 121-124, 1944.
22. Koritz SB and Cohen PP: *J Biol Chem.* **243**: 3924, 1968.
23. Kampf A, Bariknecht R, Schaffer P, Osaki S and Mertes MP: *J. Med Chem.* **19**: 903-908, 1976.
24. Ho YK, Hakala T, and Zakrzewski S: *Cancer Res.* **32**: 1023-1028, 1972.
25. Spassova MK, Russev GC and Goovinsky EV: *Biochem Pharmacol.* **25**: 923-924, 1976.
26. Becker JH and Lohr GW: *Klin Wochenschr.* **57**: 1109-1115, 1979.
27. Bagnara AS and Finch LR: *Anal. Biochem.* **45**: 24-28, 1971.
28. Hunting D and Henderson J: *Can J Biochem.* **59**: 723-727,1981.
29. Suzuki H, Nishimura T, Muto SK and Tanaka N: *J Antibacteriol.* **32**: 875-883, 1978.
30. Pera JF Sr, Rawlings CJ, Shackleton J and Robert JJ: *Biochem. Biophy. Acta.* **655**:152-166, 1981.
31. Woynarowski JW, Beerman TA and Konopa J: *Biochem. Pharmacol.* **30**: 3005-3007, 1981.
32. Zhao Y, Hall IH, Oswald CB, Yokoi T and Lee KH: *Chem. Pharm. Bull.* **35**: 2052-2061, 1987.
33. Kunchandy E and Rao MNA: *Int'l J. Pharmaceutics* **57**: 173-176, 1989.
34. Roch-Arveiller M, Revelant V, Pham Huy D, Maman L, Fontagne J, Sorenson JRJ, and Giroud JP: *Agents and Actions* **31**: 65-70, 1990.
35. Stein CA, LaRocca RV, ThomasR, McAtee N and Myers CE: J. Clinical Oncol. 7:499-508, 1989.
36. La Rocca R.V, Meer J, Gilliatt R. W, Stein CA, Cassidy J, Myers CE and ZDAlakas MC : *Neurology* **40**: 954-960, 1990.
37. Myers C, Cooper M, Stein A, LaRocca R, Walther MM, Weiss G, Choyke P, Dawson N, Steinberg S, Uhrich MM, Cassidy J, Kohler DR, Trepel J and Linehan M: *J. Clin. Oncol.* **10**: 881-889, 1992.
38. Jindal HK, Anderson CW, Davis RG and Vishwanatha JK: *Cancer Res.* **50**: 7754-7757, 1990.
39. Funayama Y, Nishio K, Takeda Y, Kubota N, Ohira T, Ohmori T, Ohta S, Ogasawara H, Hasegawa S and Saijo N: *Anticancer Res.* **13**: 1981-1988, 1993.
41. Bojanowski K, Lelievre S, Markovits J, Couprie J, Jacquemin-Sablon A and Larson AK:. *Proc Natl Acad Sci. USA* **89**: 3025-3029, 1992.
42. Ono K, Nakane H and Fukushima M:*Eur. J. Biochem.* **172**, 349-353, 1988.
43. Lopez RL, van Rijswijk REN, Wagstaff J, Pinedo HM and Peters GJ: *Eur. J. Cancer* **30**A: 1545-1549, 1994.

**Received: April 2, 1998 - Accepted: April 24, 1998 -**
**Received in revised camera-ready format: April 29, 1998**

